# Peptide nucleic acid conjugates and their antimicrobial applications—a mini-review

**DOI:** 10.1007/s00249-023-01673-w

**Published:** 2023-08-23

**Authors:** Uladzislava Tsylents, Izabela Siekierska, Joanna Trylska

**Affiliations:** Centre of New Technologies, University of Warsaw, Banacha 2C, 02-097 Warsaw, Poland

**Keywords:** Peptide nucleic acid (PNA), Antisense oligonucleotides, Antimicrobial activity, Cell-penetrating peptides, Aminoglycosides, Vitamin B_12_

## Abstract

Peptide nucleic acid (PNA) is a nucleic acid mimic with high specificity and binding affinity to natural DNA or RNA, as well as resistance to enzymatic degradation. PNA sequences can be designed to selectively silence gene expression, which makes PNA a promising tool for antimicrobial applications. However, the poor membrane permeability of PNA remains the main limiting factor for its applications in cells. To overcome this obstacle, PNA conjugates with different molecules have been developed. This mini-review focuses on covalently linked conjugates of PNA with cell-penetrating peptides, aminosugars, aminoglycoside antibiotics, and non-peptidic molecules that were tested, primarily as PNA carriers, in antibacterial and antiviral applications. The chemistries of the conjugation and the applied linkers are also discussed.

## Introduction

**Fig. 1:**
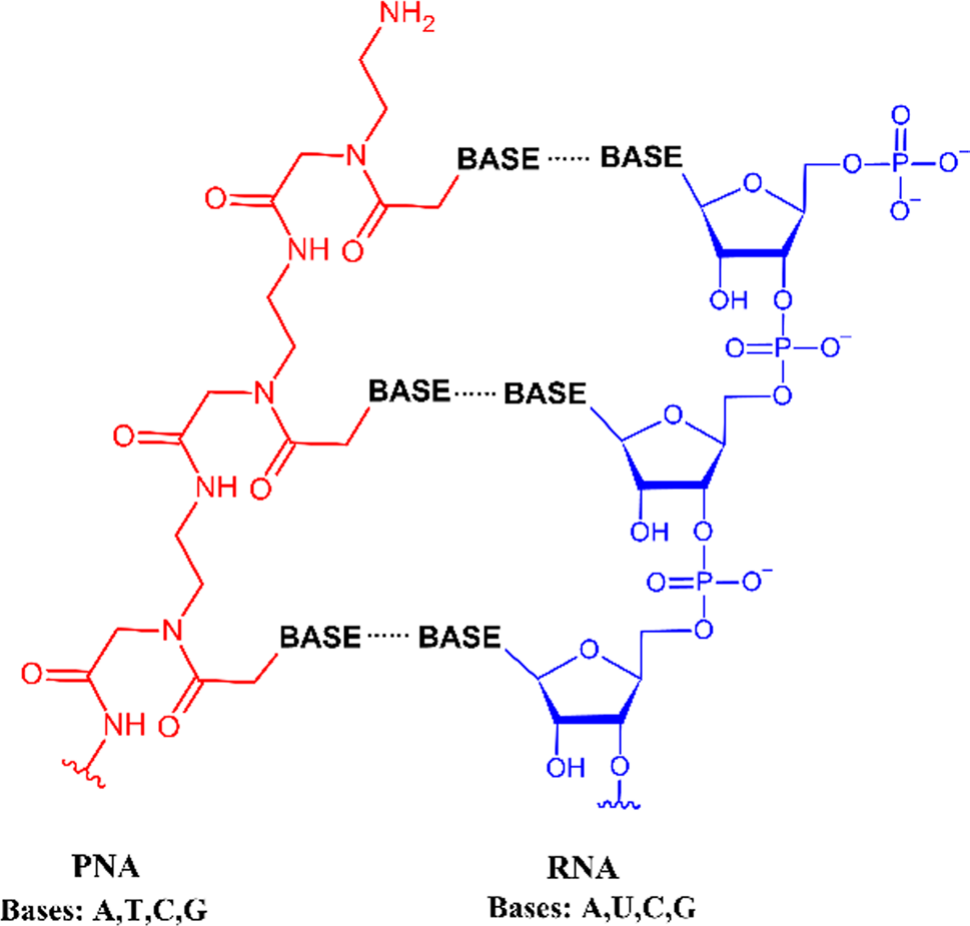
Comparison of the PNA and RNA structures. The ribose-phosphate backbone of RNA (blue) is replaced by N-(2-aminoethyl)-glycine units in PNA (red)

Peptide nucleic acid (PNA) is a synthetic DNA analog, first synthesized in 1991, with a peptide-like backbone composed of N-(2-aminoethyl)-glycine units (Nielsen et al. 1991). The distances between nucleobases in PNA oligomers resemble the ones in DNA or RNA. In addition, thanks to its charge-neutral backbone, PNA binds to natural nucleic acid oligomers with high affinity and forms thermally stable duplexes (Fig. 1).

Short PNA oligomers are of vast interest in molecular biology, biotechnology, diagnostics, and medicine because they can be applied as antigene and antisense agents (Montazersaheb et al. 2018; Abdi et al. 2020; Singh et al. 2020). The antigene PNA oligomers recognize and bind complementary DNA fragments of a specific gene and interfere with its transcription. By forming a triplex with DNA or by strand invasion of a DNA duplex, PNA can block the activity of the RNA polymerase (Jakob Larsen and Nielsen 1996; D’Souza et al. 2018). In antisense strategies, PNA hybridizes with various kinds of RNA and hinders RNA processing, transport into the cytoplasm, or translation (Montazersaheb et al. 2018; Singh et al. 2020; Brazil 2023). PNA does not induce RNA cleavage by ribonuclease H but sterically blocks the RNA target. Since PNA is a peptide–nucleic acid hybrid, it is not recognized by nucleases and proteases, which makes it biostable. In comparison with other oligonucleotides, PNA oligomers require only 9 to 12 monomers to effectively discriminate and efficiently bind the target (Goltermann et al. 2019; Popella et al. 2022).

However, the common use of PNA oligomers is limited due to their poor water solubility and lack of membrane permeability precluding their cellular uptake (Kumar and Ganesh 2005). These drawbacks are being addressed by various PNA modifications (Wojciechowska et al. 2020; Brodyagin et al. 2021; Pradeep et al. 2023). Solubility depends on the PNA sequence, i.e., aggregation may occur for sequences “overloaded” with purines (with guanines being more problematic than adenines). Nevertheless, the solubility can be improved by nucleobase and backbone modifications or simply by attaching lysine or polyamines at the PNA terminus (Brodyagin et al. 2021).

**Fig. 2:**
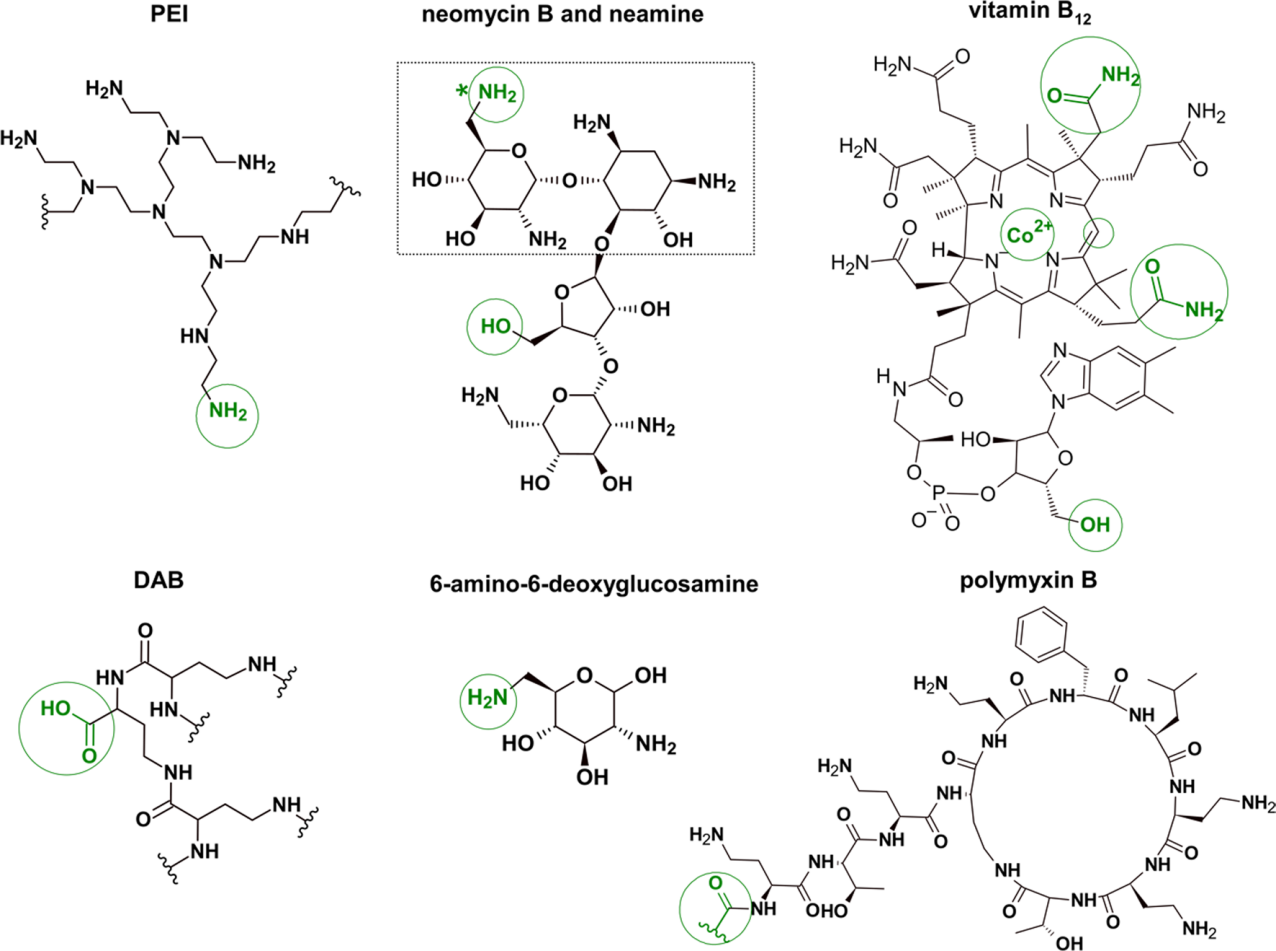
Schematic overview of various components of the PNA conjugates that were tested in antiviral or antibacterial applications. The PNA attachment points are in green. The asterisk (*) marks the PNA conjugation site only for neamine (which is framed)

Different approaches to enhance the cellular uptake of PNA have been developed (Nielsen 1999; Gambari 2001; Mehiri et al. 2008). Cellular permeability can be achieved through chemical modifications, e.g., by introducing amino acids into the PNA backbone (γ-modified PNA), which are thoroughly described in these reviews (Brodyagin et al. 2021; Brazil 2023; Pradeep et al. 2023). Moreover, PNA has been covalently linked to miscellaneous carrier molecules, among which the cell-penetrating peptides (CPPs) are the most common but non-peptidic vectors have been also developed (Fig. 2). Many PNA carriers have been tested and one was effective enough to make the PNA-based strategy reach a clinical stage (EOM Pharmaceuticals 2022; Rádis-Baptista et al. 2017; Gasparello et al. 2022). Other delivery systems based on nanoparticles (Malik et al. 2019; Oyaghire et al. 2020), liposome formulations (Löffler et al. 2020), self-assembling capsid proteins (Macadangdang et al. 2011), and the DNA tetrahedron (Zhang et al. 2018) were also described (Volpi et al. 2021).

Several recent studies and reviews on PNA-based conjugates in biomedical applications (Brodyagin et al. 2021) including gene-editing (Economos et al. 2020; Brazil 2023), PNA-FISH assays (Almeida et al. 2011; Huang et al. 2019), antibacterials (Wojciechowska et al. 2020), and diagnostics (Pradeep et al. 2023) were published. Therefore, this mini-review focuses exclusively on PNA conjugates in which PNA is covalently linked with either carriers (such as CPPs and small non-peptidic molecules) or aminoglycoside antibiotics including their constituents. We describe synthetic approaches used to obtain the linkages and provide examples of applications of these PNA conjugates targeted at bacterial and viral RNA.

## Conjugation of PNA with peptides

To overcome poor membrane permeability and cellular uptake of PNA oligomers, PNA conjugation with CPPs was proposed (Good et al. 2001). CPPs are promising PNA carriers because they can destabilize or translocate through the bacterial membrane (Rádis-Baptista et al. 2017). Also, CPPs typically have a positive net charge, which attracts the PNA conjugate to its negatively charged DNA or RNA target. The effectiveness of CPPs conjugated to PNA was typically quantified by the antibacterial activity of CPP–PNA, i.e., by determining the minimal inhibitory concentration (MIC) of the conjugate required to inhibit bacterial growth, with the PNA sequence targeting the transcript of an essential gene (Hatamoto et al. 2010). The antibacterial activities of the further described CPP–PNA conjugates are gathered in Table 1.

**Table 1:** Overview of the applications of the antimicrobial PNA-based conjugates including mRNA targets and organisms

Conjugate	PNA target	Target organism	Linkage	MIC (µM)	Reference
(KFF)_3_K-PNA	*acpP*	*E. coli* K-12	eg1	0.5-1	Good et al. (2001); Goltermann et al. (2019)
	*acpP*	*E. coli* K-12	triazole ring	5	Równicki et al. (2019)
	*acpP*	*E. coli* ESBL	eg1	6.25	Bai et al. (2012)
	*acpP*	*E. coli* MDR	eg1	25	Bai et al. (2012)
	*acpP*	*E. coli ΔSbmA*	eg1	8-16	Goltermann et al. (2019)
	*acpP*	*E. coli* LPS mutants	eg1	0.03-1	Goltermann et al. (2019, 2022)
	*acpP*	*E. coli* Evo1-3 mutants	eg1	0.25	Frimodt-Møller et al. (2021)
	*acpP*	*E. coli* BW25113 mutants	eg1	0.125-1	Goltermann et al. (2022)
	*acpP*	UPEC	–	1.25	Popella et al. (2022)
	*acpP*	*S. enterica* serovar Typhimurium SL1344	–	5	Popella et al. (2021)
	*acpP*	*K. pneumonia* ATCC 13883	eg1	2	Goltermann et al. (2019)
	*acpP*	*K. pneumoniae* ESBL	eg1	40	Bai et al. (2012)
	*acpP*	*S. enterica* MDR	eg1	25	Bai et al. (2012)
	*acpP*	*S. flexneri* MDR	eg1	5	Bai et al. (2012)
	*gyrA*	*S. pyogenes* M49	eg1	10	Patenge et al. (2013)
	*rpoA*	*L. monocytogenes* clinical isolates	–	2-32	Abushahba et al. (2016)
	*dnaB*	UPEC	–	5	Popella et al. (2022)
	*ftsZ*	UPEC	–	2.5	Popella et al. (2022)
	*rpsH*	UPEC	–	2.5	Popella et al. (2022)
	*mazE*	*E. coli* K-12	eg1	16	Równicki et al. (2018)
	*mazE*	*E. coli* O157:H7	eg1	16	Równicki et al. (2018)
	*mazE*	*E. coli* WR 3551/98	eg1	16	Równicki et al. (2018)
	*hipB*	*E. coli* K-12	eg1	8	Równicki et al. (2018)
	*hipB*	*E. coli* O157:H7	eg1	>16	Równicki et al. (2018)
	*hipB*	*E. coli* WR 3551/98	eg1	16	Równicki et al. (2018)
	*thyA*	*E. coli* K-12	eg1	>16	Równicki et al. (2018)
	*thyA*	*E. coli* O157:H7	eg1	16	Równicki et al. (2018)
	*thyA*	*E. coli* WR 3551/98	eg1	16	Równicki et al. (2018)
	*gltX*	*E. coli* K-12	eg1	4	Równicki et al. (2018)
	*gltX*	*E. coli* O157:H7	eg1	16	Równicki et al. (2018)
	*gltX*	*E. coli* WR 3551/98	eg1	1	Równicki et al. (2018)
TAT-PNA	*acpP*	UPEC	–	5	Popella et al. (2022)
	*acpP*	*S. enterica* serovar Typhimurium SL1344	–	10	Popella et al. (2021)
	*gyrA*	*S. pyogenes* M49	eg1	1	Patenge et al. (2013)
	*gyrA*	*S. pyogenes* M49	eg1	15.6	Barkowsky et al. (2019)
	*rpoA*	*L. monocytogenes* clinical isolates	–	0.5-4	Abushahba et al. (2016)
(RXR)_4_XB-PNA	*acpP*	*E. coli* K-12	amide bond	1-2	Goltermann et al. (2019, 2022)
	*acpP*	*E. coli* ESBLs	eg1	5	(Bai et al. 2012)
	*acpP*	*E. coli* MDR	eg1	25	(Bai et al. 2012)
	*acpP*	*E. coli ΔSbmA*	amide bond	1	Goltermann et al. (2019)
	*acpP*	*E. coli* LPS mutants	amide bond	0.25-4	Goltermann et al. (2019, 2022)
	*acpP*	*E. coli* Evo mutants	amide bond	8	Frimodt-Møller et al. (2021)
	*acpP*	*E. coli* BW25113 mutants	amide bond	0.5-2	Goltermann et al. (2022)
	*acpP*	*S. enterica* serovar Typhimurium SL1344	–	5	Popella et al. (2021)
	*acpP*	*K. pneumoniae* ESBLs	eg1	30	Bai et al. (2012)
	*acpP*	*S. enteric* MDR	eg1	12.5	Bai et al. (2012)
	*acpP*	*S. flexneri* MDR	eg1	2.5	Bai et al. (2012)
	*gyrA*	*S. pyogenes* M49	eg1	62.5	Barkowsky et al. (2019)
	*rpoA*	*L. monocytogenes* clinical isolates	–	0.25-4	Abushahba et al. (2016)
	*mraY*	*P. aeruginosa* PAO1	eg1	–	Maekawa et al. (2015)
	*lepB*	*P. aeruginosa* PAO1	eg1	–	Maekawa et al. (2015)
	*lptD*	*P. aeruginosa* PAO1	eg1	–	Maekawa et al. (2015)
(RX)_6_B-PNA	*acpP* *acpP* *acpP* *acpP*	*E. coli* K-12 *E. coli ΔSbmA* *E. coli* LPS mutants *E. coli* BW25113 mutants	amide bond amide bond amide bond amide bond	1 1 0.25-4 0.5-2	Goltermann et al. (2019) Goltermann et al. (2019) Goltermann et al. (2019, 2022) Goltermann et al. (2022)
Transportan-PNA	TAR RNA	HIV-1	disulfide bridge	–	Turner et al. (2005)
R_6_-Penetratin-PNA	TAR RNA	HIV-1	disulfide bridge	–	Turner et al. (2005)
(R/W)9-PNA	PPT RNA	HIV-1	disulfide bridge	–	Cordier et al. (2014)
	PPT RNA	HIV-1	maleimide linker	–	Cordier et al. (2014)
BF2-A-PNA	*acpP*	*E. coli* K-12	SMCC	0.9	Hansen et al. (2016)
Drosocin-PNA	*acpP*	*E. coli* K-12	SMCC	0.9	Hansen et al. (2016)
Oncocin 10-PNA	*acpP*	*E. coli* K-12	SMCC	0.9	Hansen et al. (2016)
BF2-A-RXR-PNA	*acpP*	*E. coli* K-12	SMCC	≤0.9	Hansen et al. (2016)
Drosocin-RXR-PNA	*acpP*	*E. coli* K-12	SMCC	3	Hansen et al. (2016)
Polymyxin-PNA	*acpP*	*E. coli* DH5α	Cys-CBT	–	Patil et al. (2022)
	*acpP*	*A. baumannii* 5075	Cys-CBT	–	Patil et al. (2022)
	*acpP*	*A. baumannii* 5075D	Cys-CBT	–	Patil et al. (2022)
	*acpP*	*K. pneumoniae*	Cys-CBT	–	Patil et al. (2022)
	*acpP*	*P. aeruginosa*	Cys-CBT	–	Patil et al. (2022)
Gbu-DAB-PNA	*acpP*	*E. coli* K-12	amide bond	0.5	Iubatti et al. (2022)
	*acpP*	*E. coli ΔSbmA*	amide bond	0.5	Iubatti et al. (2022)
	*acpP*	*K. pneumoniae*	amide bond	8	Iubatti et al. (2022)
	*ftsZ*	*E. coli* K-12	amide bond	2	Iubatti et al. (2022)
	*ftsZ*	*E. coli ΔSbmA*	amide bond	4	Iubatti et al. (2022)
	*ftsZ*	*K. pneumoniae*	amide bond	16	Iubatti et al. (2022)
Goc-DAB-PNA	*acpP*	*E. coli* K-12	amide bond	0.25	Iubatti et al. (2022)
	*acpP*	*E. coli ΔSbmA*	amide bond	0.25	Iubatti et al. (2022)
	*acpP*	*K. pneumoniae*	amide bond	0.125	Iubatti et al. (2022)
Vitamin B_12_-PNA	*acpP*	*E. coli* K-12	triazole ring	5	Równicki et al. (2019)
Neamine-PNA	TAR RNA	HIV-1	amide bond	1*	Riguet et al. (2004)
Aminoglucosamine-PNA	TAR RNA	HIV-1	amide bond	0.8*	Das et al. (2012)

The conjugation technique is presented by the type of linker or bond between the PNA and the conjugated compound

*IC_50_ values; “– “no information or not determined

*acpP* - acyl carrier protein gene, ESBL - extended spectrum beta-lactamase, MDR - multidrug resistance, *SbmA* - inner-membrane peptide transporter gene, LPS lipopolysaccharide, Evo1-3 mutants - selected clones with Cpx-envelope stress response system-based resistance, UPEC - uropathogenic *Escherichia coli*, *gyrA* - the DNA topoisomerase gyrase subunit A gene, *rpoA* - the RNA polymerase α subunit gene, *dnaB* - replicative DNA helicase gene, *ftsZ* - cell division protein gene, *rpsH* - 30S ribosomal subunit protein S8 gene, *mazE* - endoribonuclease antitoxin protein gene, *hipB* - glutamyl-tRNA synthetase antitoxin protein gene, *thyA* - thymidylate synthase protein gene, *gltX* - glutamyl-tRNA synthetase gene, X - 6-aminohexanoic acid, B - β-alanine, *mraY* - undecaprenyl-phosphate phospho-N-acetylmuramoyl-pentapeptide transferase gene, *lepB* - type I signal peptidase gene, *lptD* - lipopolysaccharide transport component gene, BF2-A - buforin 2-A, SMCC - succinimidyl 4-(*N*-maleimidomethyl)-cyclohexane-1-carboxylate, TAR RNA - binding site of the viral protein TAT modulating the transcription of the HIV genome, Gbu - guanidinobutanoyl, Goc - guanidinooctanoyl

**Fig. 3:**
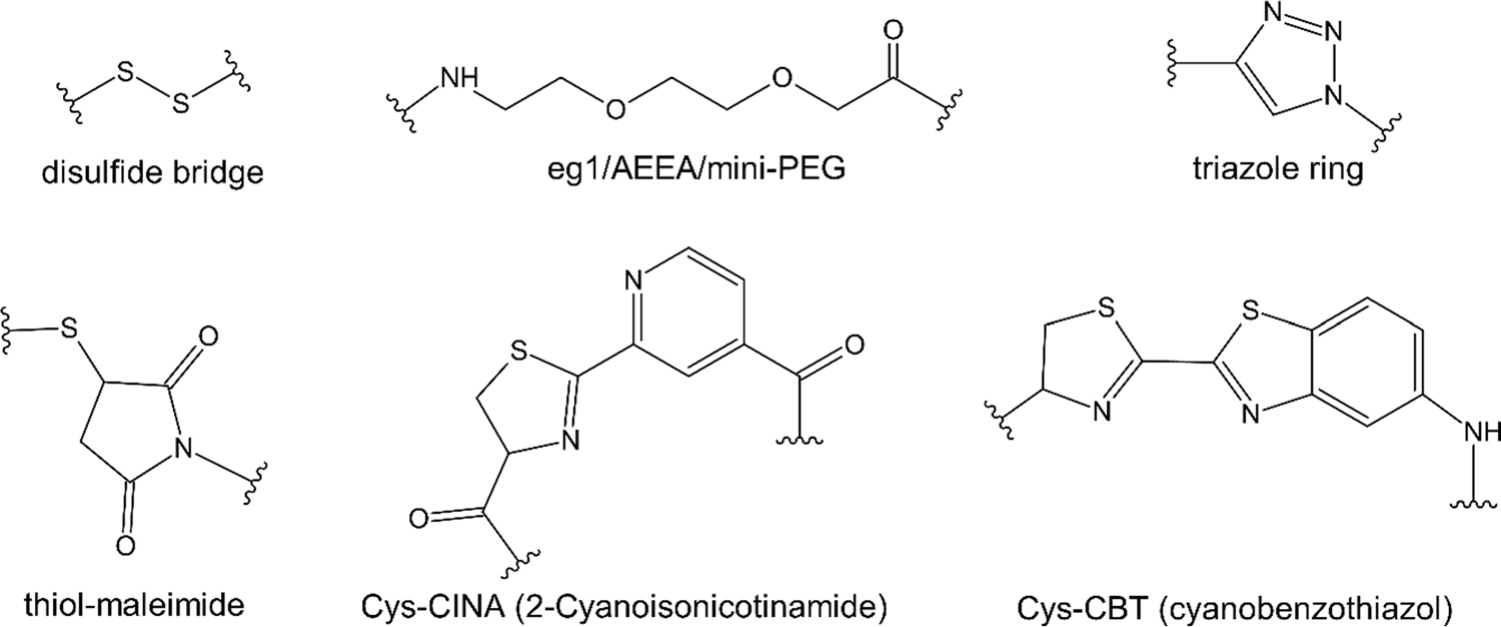
Linkers used for PNA conjugation

General synthetic approaches for the conjugation of CPPs and various oligonucleotides, not only PNA, are thoroughly reviewed in (Klabenkova et al. 2021). The commonly used linkers (see Fig. 3), applicable for PNA conjugation, are either the non-degradable ethylene glycol (eg1), also known as 2-aminoethoxy-2-ethoxyacetic acid (AEEA) or mini-PEG, or the in-cell degradable disulfide bond. The eg1 linker is attached to the N-terminus of PNA on the solid support. To obtain degradable disulfide linkers, direct oxidation of thiol groups can be performed. Either the thiol-containing fragments can be activated (Rentier et al. 2017) or the S-sulfonate-protected cysteine can be used (Dirin et al. 2015). Other frequent synthetic strategies to link CPP with PNA include 1,3-dipolar cycloaddition to form a triazole ring or maleimide moiety via the Diels–Alder reaction (Brun et al. 2015). However, most techniques require orthogonal protecting groups and their application is limited to linear peptides.

The conjugation of PNA with structurally more complex compounds like the membrane-active cyclic peptide polymyxin required unusual linkage strategies, so a different conjugation method was developed (Patil et al. 2019, 2022). Patil et al. explored the 2-cyanothiazole (CBT) approach and compared it with other conjugation methods like the alkyne-azide click reaction (Fig. 3). They also applied the 2-cyanoisonicotinamide (CINA) group for low-cost and efficient synthesis of the PNA-based conjugates.

### The lysine-rich (KFF)_3_K peptide

A frequently used peptide, covalently linked with PNA, is a short CPP composed of lysines and phenylalanines. (KFF)_3_K is a linear, cationic and amphipathic peptide used as an antibiotic enhancer (Vaara and Porro 1996), because it facilitates the uptake of many antimicrobial compounds by destabilizing the outer membrane. Conjugation of (KFF)_3_K with a PNA oligomer targeting the essential acyl carrier protein gene, *acpP*, resulted in antibacterial activity in *E. coli* with MIC of 1 µM (Good et al. 2001). Following this, (KFF)_3_K became one of the commonly used peptides for PNA delivery to Gram-negative and even Gram-positive bacteria (Kulyté et al. 2005; Patenge et al. 2013; Abushahba et al. 2016). The optimal PNA sequence length in the (KFF)_3_K–PNA conjugates was found to be 10–12 monomers (Goltermann et al. 2019) in the case of the *acpP* target and *E. coli* cells. Other PNA targets encoding essential genes, such as *dnaB* (replicative DNA helicase gene), *ftsZ* (cell division protein gene), *rpsH* (30S ribosomal subunit protein S8 gene), were recently tested showing that even 9-mer PNAs provide similar or even higher antibacterial effect in the uropathogenic strain of *E. coli* (UPEC) (Popella et al. 2022). The delivery properties of the (KFF)_3_K peptide were also confirmed for PNA sequences that were designed for non-essential targets such as toxin–antitoxin systems (Równicki et al. 2018).

Unfortunately, the most limiting factor for effective silencing activity of the (KFF)_3_K–PNA conjugates remains the stability of the peptide itself. It was shown that the half-life of (KFF)_3_K in bacterial cell culture is 1 h and drops below 5 min in the presence of peptidases (Yavari et al. 2021). To increase the proteolytic stability of the (KFF)_3_K peptide, the d-form (KFF)_3_K was used in a conjugate with PNA targeting the *acpP* essential gene (Ghosal et al. 2013; Yavari et al. 2021). Even though the d-(KFF)_3_K conjugates with PNA showed four times lower antimicrobial activity in the *E. coli* MG1655 strain than the l-isomer, their stability was overall improved. Additionally, as recently shown, the full-length d-(KFF)_3_K conjugate crossed the membrane independently of the SbmA inner-membrane peptide transporter (Yavari et al. 2021; Frimodt-Møller et al. 2022). The previously described dependence of the l-form of this peptide on the SbmA receptor (Puckett et al. 2012; Ghosal et al. 2013) is rather a consequence of the rapid degradation of l-(KFF)_3_K. However, although useful in laboratory research, the medical application of PNA conjugates with (KFF)_3_K is rather doubtful due to the hemolytic side effects of this peptide (Vaara and Porro 1996). Recently, the hemolytic activity of the (KFF)_3_K–PNA conjugate of 21% was observed on mice erythrocytes at the concentration of 50 µM needed to inhibit bacterial growth of *K. pneumoniae* (da Silva et al. 2021).

### Arginine-containing peptides

Another peptide carrier of PNA, the trans-activator of transcription (TAT) peptide, was discovered by two laboratories during research on the human immunodeficiency virus (HIV) (Frankel and Pabo 1988; Green and Loewenstein 1988). The TAT peptide, with the sequence GRKKRRQRRR, is a fragment of an RNA-binding TAT protein essential in HIV-1 replication. The cell-penetrating nature of TAT comes from the six Arg and two Lys residues that allow the uptake of TAT and its conjugates by various cell types (Herce and Garcia 2007; Kanwar et al. 2012). The efficacy of gene silencing by PNA conjugates with TAT and other arginine-based peptides was compared with the (KFF)_3_K–PNA conjugates on many bacterial strains such as *E. coli* (Bai et al. 2012; Goltermann et al. 2019)*, S. enterica* serovar Typhimurum (Popella et al. 2021), S. *pyogenes* (Patenge et al. 2013; Barkowsky et al. 2019), *L. monocytogenes* (Abushahba et al. 2016), *P. aeruginosa* (Maekawa et al. 2015) (Table 1). In *S. pyogenes*, the PNA conjugates with the TAT peptide showed only slightly improved gene silencing effect, resulting in PNA-induced growth inhibition, as compared to (KFF)_3_K–PNA (at concentrations of 0.4–1.6 µM for TAT vs. 1.6–4.0 µM for (KFF)_3_K (Patenge et al. 2013)). However, in some *L. monocytogenes* strains, 16-fold improvements in antibacterial activity were reported (with MIC of 2 µM for TAT–PNA vs. 32 µM for (KFF)_3_K–PNA (Abushahba et al. 2016)).

The discovery of the importance of arginines in CPPs led to finding the optimal R-X-R (X—6-aminohexanoic acid) motif and introducing the (RXR)_4_XB (B—β-alanine) peptide (Nelson et al. 2005; Abes et al. 2006). In an in vitro killing assay, the (RXR)_4_XB–PNA conjugates showed almost two times higher bactericidal activity (1.60 vs 3.18 Δlog decrease in colony forming units (CFU)) on *S. pneumoniae* strains than TAT–PNA conjugates (Barkowsky et al. 2022). Contrary, on *S. pyogenes*, the MIC values of these two conjugates (Barkowsky et al. 2019) were four times lower for TAT–PNA (15.6 µM) than for (RXR)_4_XB–PNA (62.5 µM). Interestingly, some *E. coli* mutants lacking proteins involved in the outer-membrane stability (namely *tolB, tolQ* and *tolR)* were more susceptible to (KFF)_3_K–PNA than to (RXR)_4_XB–PNA (Goltermann et al. 2022). On the other hand, the *E. coli* strains with different carbohydrate structures of the outer core of lipopolysaccharides (R3 and R4 mutants) showed two to four times higher susceptibility toward arginine-based conjugates with PNA (based on the MIC values) than those without arginine (Goltermann et al. 2022). Further, a potential resistance mechanism to arginine-based CPP––PNA conjugates was investigated in *E. coli* mutants with constitutively activated Cpx-envelope stress response, which decreases the inner-membrane potential (Frimodt-Møller et al. 2021). Higher resistance toward the (RXR)_4_XB–PNA conjugates was observed based on MIC of 8 µM (as compared to 0.5 µM for the wild-type strain). These findings confirmed the importance of the membrane electric potential for the arginine-rich peptides used for PNA uptake.

The above examples included arginine-rich peptides covalently linked to PNA typically using the eg1 linker (Bai et al. 2012; Patenge et al. 2013; Maekawa et al. 2015) or directly via an amide bond (Goltermann et al. 2019, 2022). Introducing a disulfide bridge as a linker into such PNA conjugates was also investigated, but antimicrobial applications are scarce (Stasińska et al. 2020). The arginine-rich peptides in disulfide-linked PNA conjugates aimed at viral RNA introduced into eukaryotic cells (Turner et al. 2005). The target RNA was the HIV-1 trans-activation responsive (TAR) element, the fragment of the genome, which is the binding site of the TAT protein. The PNA conjugates targeted the TAR RNA positioned upstream of the firefly luciferase gene as a reporter protein in the HeLa cell line. The eg1-linked PNA conjugates did not significantly reduce the luciferase expression at concentrations up to 2.5 µM. However, the R_6_-penetratin and transportan, which were disulfide linked to PNA, showed a 60% decrease in luciferase activity after 24 h (Turner et al. 2005). Another arginine-rich RRWWRRWRR peptide, known as (R/W)9, was conjugated to the PNA via a disulfide bond or maleimide linker, and later tested in a similar assay in HeLa cells with luciferase reporter (Cordier et al. 2014). The PNA sequence used in this study targeted the HIV-1 polypurine tract (PPT). This PPT RNA is used as a primer for the plus-strand DNA synthesis, which makes PPT essential for efficient replication of most retroviruses (Miles et al. 2005). In the presence of the chloroquine antibiotic, both PNA conjugates with (R/W)9 (at concentrations of 0.5–1.5 µM) inhibited luciferase activity in a dose-dependent manner. However, at 1.5 µM, the disulfide-linked (R/W)9-SS–PNA conjugate was less effective (with a 68% ± 8 decrease in luciferase activity) than the maleimide-linked (R/W)9–PNA (with an 85% ± 4 decrease).

### Antimicrobial peptides

Although research on peptides that can deliver PNA to cells focused mostly on the cationic (KFF)_3_K or repetitive arginine-based motifs, antibacterial peptides, but with intracellular targets, were also conjugated to PNA and tested (Hansen et al. 2016). The authors synthesized PNA-based conjugates with arginine- and lysine-containing antimicrobial peptides (AMPs) in a thiol-maleimide reaction resulting in compounds with the succinimidyl 4-(*N*-maleimidomethyl)-cyclohexane-1-carboxylate (SMCC) linker. Some of these conjugates were found to deliver PNA targeting the essential *acpP* transcript to *E. coli* cells (with MIC values of the conjugates, resulting from PNA inhibition, in the range of 0.9–4 µM). Additionally, the dependence of this delivery on the SbmA inner-membrane peptide transporter was investigated. PNA conjugates with the peptides buforin 2-A (BF2-A), drosocin, oncocin 10, and BF2-A-RXR showed the lowest MIC of ≤ 0.9 µM, but their activity depended on SbmA. The PNA conjugate with drosocin was the most prominent PNA carrier, with the highest difference in MIC values between the conjugate and unconjugated peptide (0.9 µM and > 33 µM, respectively). In the case of SbmA-independent PNA carriers, the best activities, with MIC of 3 µM, were reported for the PNA conjugated with drosocin-RXR (with MIC > 10 µM for the unconjugated peptide). These observations encourage further investigation of not only CPPs as PNA carriers but also AMPs that act inside cells.

## Non-peptidic molecules as PNA carriers

### Conjugation with cationic polymers

Although research on improving PNA solubility and cellular uptake has been dominated by PNA conjugates with CPPs, other strategies based on non-peptidic compounds were tested (mostly in eukaryotic but also bacterial cells). For example, PNA was conjugated to polyethyleneimine (PEI, Fig. 2) (Berthold et al. 2010). PEI is a common cationic polymer that can deliver oligonucleotides via an endocytotic pathway (Boussif et al. 1995). The PEI–PNA conjugate was obtained using a thiol-containing linker to form a cleavable complex with cysteine-containing PNA. The splice correction assay in HeLa cells (Kang et al. 1998) used a PNA sequence that targeted the aberrant splice site, and as an effect corrected splicing and enabled the measurements of the luciferase activity as a reporter protein. The luciferase activity of one of the most potent PEI–PNA sequences was about tenfold higher than for R_8_–PNA. Recently, the same group tested another cationic compound, diaminobutanoic acid (DAB) called dendron (Fig. 2), as a PNA carrier to Gram-negative bacteria (Iubatti et al. 2022). They also investigated the effect of the carbon chain extension in DAB. DAB–PNA conjugates with guanidinoctanoic (Goc) and guanidinobutanoic (Gbu) acid showed promising MIC values (in both wild type and Δ*sbmA E. coli* strains) of 0.25 and 0.5 µM, respectively, (Table 1). In addition, their toxicity to HepG2 cells was negligible at least up to the concentration 100-fold higher than MIC. The in vivo efficacy against multidrug-resistant *E. coli* clinical isolates made the authors suggest that these new DAB dendrons might serve as a bacterial delivery platform for PNA with potential in vivo use.

### PNA conjugates with vitamin B_12_

Our group showed that vitamin B_12_ (an essential nutrient for most bacteria, Fig. 2) transports PNA into *E. coli* and *S.* Typhimurium cells (Równicki et al. 2017). We synthesized a series of vitamin B_12_–PNA conjugates to evaluate if the linker type and length affect bacterial uptake of the conjugates. Non-cleavable linkers were formed in 1,3-dipolar cycloaddition, while the cleavable disulfide bridge was obtained by conjugation of Cys-containing PNA with B_12_-SS-pyridyl derivative (Wierzba et al. 2016; Równicki et al. 2017). All the conjugates were delivered to the cytoplasm, but the highest potential to block translation of the *mrfp1* mRNA (the reporter gene expressing Red Fluorescent Protein) was observed for the conjugates bearing either the shortest linker (with triazole ring directly connected to the 5’-OH position of vitamin B_12_) or the longest spacer (containing 12 carbon atoms). Also, the attachment point of PNA to vitamin B_12_ was tested to evaluate if it affects PNA transport (Wierzba et al. 2018). It turned out that vitamin B_12_ delivered PNA with the highest efficiency if it was attached to the 5’-OH position (Fig. 2). Using the PNA sequence targeting the *acpP* transcript, we also found that PNA conjugates with vitamin B_12_ inhibited *E. coli* growth, but the inhibition efficiency was lower than for the corresponding PNA conjugated to (KFF)_3_K (Równicki et al. 2019). Nevertheless, these studies have shown that the receptor-based and energy-dependent transport can be hijacked to deliver PNA to Gram-negative bacterial cells and served as the proof-of-concept for the Trojan horse strategy for PNA. PNA has advantages over charged nucleic acids in terms of receptor-specific transport because PNA can permeate through the hydrophobic membrane receptors (Pieńko et al. 2021). Delivery of 2’O-methyl RNA oligomers conjugated to vitamin B_12_ to Gram-negative bacteria was less effective than PNA (Giedyk et al. 2019).

## PNA conjugates with aminosugars

An idea to conjugate PNA to aminoglycosides (AGs) or their constituents—aminosugars—was also explored. However, contrary to the above-described peptide–PNA conjugates with antibacterial properties, so far only the antiviral RNA silencing activity of PNA conjugated to various aminosugars was explored. AGs are therapeutically useful antibiotics used for over seven decades, since the discovery of streptomycin by Selman Waksman (Schatz et al. 1944). Structurally, AGs consist of two or more aminosugars connected via a glycosidic bond to the central hexose or aminocyclitol. Due to cationic amino groups, these positively charged polysaccharides efficiently bind to ribosomal RNA in the 30S subunit and impair protein synthesis in bacteria (Magnet and Blanchard 2005). In antiviral applications, AGs may target many crucial stages of the viral life cycle, e.g., entry to the host cell or gene transcription by affecting regulatory RNAs such as HIV-1 TAR (Lapidot et al. 2008).

Initially, PNA conjugates with aminosugars were obtained to improve the binding of AGs to a specific DNA or RNA site (Charles and Arya 2005), however, increasing the solubility of PNA and promoting its cellular uptake was also considered (Riguet et al. 2004). First aminosugar–PNA conjugates were designed to target the HIV-1 TAR RNA (Riguet et al. 2004). The authors attached neamine (Fig. 2) to a 16-mer PNA using succinic anhydride with a carboxylate group necessary for the coupling reaction in the solid-phase synthesis. Measurements of firefly luciferase activity in the human lymphoblastic CEM cells, infected with virions carrying the luciferase reporter gene, showed that the neamine–PNA conjugate inhibited viral replication (with IC_50_ of about 1 µM), contrary to the naked PNA (Table 1). Therefore, this work confirmed that conjugation of PNA to neamine allows for cellular uptake of PNA which reached and blocked its specific TAR RNA target.

To understand the effects of PNA conjugation on the interactions between AG–PNAs and their target RNAs, hybrids of neamine with either RNA dinucleotides or two PNA monomers were synthesized. These conjugates were connected via an aliphatic diamine. Their binding affinity to different RNA targets (16S and 18S ribosomal RNA and TAR RNA) was determined using surface plasmon resonance (Mei et al. 2008). The neamine–PNA(T–T) conjugates exhibited twofold higher binding affinities to 18S ribosomal RNA and TAR RNA fragments than neamine alone. Moreover, molecular modeling suggested that the neamine conjugates with PNA (T–T) interacted better with RNA targets than neamine linked with RNA dinucleotides.

Despite the potency of neamine–PNA conjugates to effectively bind RNA, the number of AG amine groups that undergo protonation in physiological conditions could cause toxicity or non-specific interactions. So, the PNA conjugates containing only one ring of the AG structure were tested. The modified aminosugar, 6-amino-6-deoxyglucosamine (Fig. 2), an analog of N-acetyl-D-glucosamine was found promising in targeting TAR RNA (Das et al. 2012). This saccharide was attached to the N-terminus of the protected anti-TAR PNA sequence via an amide bond, and, subsequently, deprotected and cleaved from the solid support. Incubation of this conjugate in 0.1 M HCl for 3 days confirmed its stability under acidic conditions, which is important in terms of oral administration. Notably, uptake by the human hepatoma-derived Huh7.5 cells showed an improved distribution of the aminoglucosamine–PNA conjugate in cells. Contrary, the CPP–PNA hybrids enter these cells mostly via endocytosis, which causes entrapment of PNA in endosomes, and therefore decreases its cellular distribution (Turner et al. 2005). However, in the end, the aminoglucosamine-based PNA conjugate inhibited viral production in HIV-1 infected cells (with IC_50_ equal to 0.8 µM) with similar efficiency as the neamine–PNA conjugate (IC_50_ = 1 µM, Table 1).

PNA was also conjugated to neomycin (Fig. 2). The solid-phase synthesis of the neomycin–PNA conjugate connected via a non-degradable thiourea linkage through a multistep AG modification was first described in 2005 (Charles and Arya 2005). This conjugate was subsequently used to target a site in the Rev Response Element (RRE), a 350-nucleotide long RNA essential for HIV-1 replication (Hyun et al. 2006). Monomers and dimers of PNA were conjugated with neomycin B via a non-cleavable dimercaptohexane linker. The binding affinity and specificity of such hybrids were evaluated by fluorescence anisotropy experiments and IC_50_ measurements in the absence and presence of a genomic RNA library. The neomycin–PNA conjugates selectively bound to RRE RNA with up to fivefold higher affinities than the parent AG. Another synthetic approach to obtain the PNA dimer with neomycin or paromomycin was applied in (Alguacil et al. 2010) who for the first time reported the synthesis of AG–PNA conjugates by a combination of Huisgen alkyne-azide cycloaddition and microwave irradiation.

## Conclusion and outlook

Although PNA was conjugated to many compounds, peptides were most common and showed the best carrier properties to various cells. Nevertheless, the effectiveness of microbial delivery of such PNA conjugates could still be improved. For example, the (KFF)_3_K–PNA conjugate, targeting a functional site in 23S ribosomal RNA, inhibits protein synthesis in a cell-free system at concentrations of 2–6 μM. However, these concentrations are seven to three times lower than the MIC values of this conjugate in *E. coli* (Kulik et al. 2017), which confirms that the bacterial envelope is a tough barrier to cross for PNA-based compounds. Finding new peptides or optimizing the structure of a known CPP (Wu et al. 2007) in a CPP–PNA conjugate could lead to more effective PNA delivery to bacteria. Cationic peptides are attracted by the negatively charged bacterial membrane and once covalently linked should also facilitate binding of the neutral and hydrophobic PNA to its RNA or DNA target. But their stability and possible hemolytic activity are a concern and should be tested. Also, the membrane activity of CPPs or AMPs and their conjugates with PNA can be strain dependent; therefore, a peptide sequence that could be applied both to Gram-negative and Gram-positive bacteria might be impossible to find.

PNA may be also attached to compounds that are recognized and taken up through bacterial membrane receptors (e.g., vitamin B_12_). However, the concentrations of such metabolites necessary for growth are typically lower than those required for PNA to exert its intracellular antibacterial effect. The metabolite uptake is naturally limited (and thus also PNA in such a conjugate) and depends on the regulation of the receptor-dependent transport in a particular strain.

Some PNA conjugates with aminosugars or AGs showed antiviral potential in human cells infected with virions or in assays with the viral RNA sites incorporated into these cells. Since AGs were originally effective antibiotics, AG–PNA conjugates could be also tested in antisense approaches to target drug-resistant bacterial strains even though their envelope may be a challenging barrier for such large conjugates compared to eukaryotic cell membranes.

A comprehensive analysis of the effects of the linkers in the PNA conjugates is missing. Nevertheless, a promising approach could be based on optimizing the connection between the PNA and conjugated compound by testing various linkers (other than the commonly used eg1, Table 1) and spacers (Turner et al. 2005; Równicki et al. 2017, 2019; Klabenkova et al. 2021). Overall, the conjugation of hydrophilic molecules to PNA improves not only its solubility but also increases PNA flexibility (Pieńko et al. 2017), which could be beneficial for PNA hybridization with RNA targets.

Currently, one PNA conjugate with immunomodulatory and antiviral properties reached phase I clinical trials (EOM Pharmaceuticals 2022), which shows promise for future antibacterial PNA conjugates. Further development of PNA conjugates focused on enhancing their cellular uptake via connection to other active agents which may bring new drug candidates. Nonetheless, at this time, PNA conjugates still require further research focused on exploring new delivery vehicles or synergistically acting components.

## Data Availability

Not applicable.
